# Concentrating Ammonia from Wastewater with Electrodialysis

**DOI:** 10.1021/acsestwater.5c00721

**Published:** 2025-08-19

**Authors:** Hyuck Joo Choi, Mohammed Tahmid, Spandan Mondal, Marta C. Hatzell

**Affiliations:** † School of Chemical and Biomolecular Engineering, 115724 Georgia Institute of Technology, 311 Ferst Drive NW, Atlanta, Georgia 30332, United States; ‡ George W. Woodruff School of Mechanical Engineering, Georgia Institute of Technology, 770 Ferst Drive NW, Atlanta, Georgia 30332, United States

**Keywords:** ammonia, fertilizer, nutrient recovery, electrodialysis, membrane scaling, wastewater, resource recovery

## Abstract

Electrodialysis (ED) is a promising technology for the recovery of ammonia from wastewater. However, separating ammonia directly from complex wastewater mixtures using ED is challenging due to membrane scaling, low selectivity, and high energy consumption. Here, we evaluate the potential of electrodialysis for ammonia recovery from simulated and real wastewater mixtures. The specific energy consumption (SEC) of electrodialysis exceeded 31 kWh/kg-N for simulated wastewater but decreased 4-fold to 7 kWh/kg-N after hardness removal. Concentration factors (CFs), the final concentration relative to the initial concentration, of NH_4_^+^ for real wastewater after ultrafiltration and for synthetic wastewater without hardness were 7.5 and 10, comparable to the CF of 9 for single-salt solutions (nonmixtures). We find that the concentrated product after ED with real and simulated synthetic wastewater includes K^+^ and Na^+^, as cation exchange membranes exhibit K^+^/NH_4_^+^ and Na^+^/NH_4_^+^ selectivities near one. Thus, if the concentrated product is directly used as an aqueous fertilizer, the resulting product will be 30/30/30 for Na^+^, K^+^, and NH_4_^+^. Finally, staged electrodialysis achieved a CF of ∼50 (2.42 N wt %) with SECs of 15.2–18.1 kWh/kg-N for synthetic wastewater without hardness, demonstrating promise for recovering ammonia from wastewater with a high concentration and low energy demand.

## Graphical abstract

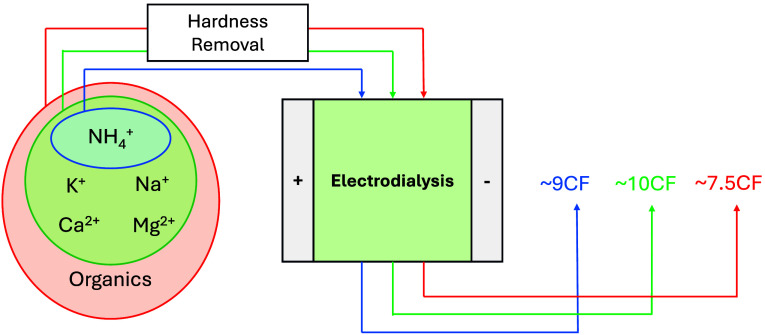

## Introduction

Nitrogen-based fertilizers (NBFs) are indispensable to meet global agricultural demands.^1,2^ Although 136 million tons of NBFs are produced annually and continue to rise,^3,4^ only 20% of applied nitrogen is absorbed by plants. The remaining 80% is lost as agricultural wastewater.^5^ Such nutrient discharges drive eutrophication and increase hazardous atmospheric emissions.^6,7^ To address these environmental and health concerns, efficient recovery and reuse of nitrogen is essential.

Electrochemical separations such as electrodialysis, capacitive deionization, and battery-electrode-based deionization have gained attention for nutrient recovery.^8–13^ Electrodialysis (ED) has also shown great potential for ammonia recovery.^14^ ED applies an electric field across ion exchange membranes, transferring ions to produce a concentrated stream (concentrate) and a dilute stream (diluate). In ED, the inorganic nitrogen nutrient is recovered in the form of ammonium (NH_4_^+^), which can be applied directly to crops such as aqua ammonia or further processed to form ammonium-based salts.^15^ Advanced ED configurations have also been investigated to recover ammonium-based nutrients, such as bipolar membrane electrodialysis (BMED)^16–18^ and selective electrodialysis (SED).^19,20^ BMED employs bipolar membranes to produce acidic and alkaline streams. Concentrating ammonium in the alkaline stream changes the equilibrium of ammonium to ammonia, allowing gaseous ammonia recovery through membrane contactors.^8,21^ BMED for ammonia recovery achieved a concentration factor (CF) of 2.5–5 and a specific energy consumption (SEC) of 3.81–25.8 kWh/kg-N.^16–18^ SED uses monovalent exchange membranes to enhance the selectivity of NH_4_^+^ while rejecting multivalent ions such as Mg^2+^ and Ca^2+^. SED generally prioritizes SEC (as low as 0.783 kWh/kg-N) and selective separation of monovalent or multivalent nutrients instead of the CF (∼1.5–2×).^19,20^

One major challenge in recovering nutrients from wastewater using ED is the complex nature of the feed stream. Wastewater can contain magnesium (5.3–150 mg/L), calcium (1.5–270 mg/L), potassium (11–789 mg/L), sodium (24–263 mg/L), and phosphorus (12–400 mg/L) in addition to nitrogen in the form of ammonia (14–4400 mg-N/L), nitrate (3.1–10 mg-N/L), and organics (23–239 mg-N/L).^22–24^ Magnesium and calcium are ionic and therefore migrate to the membrane interface when under an electric field, resulting in membrane scaling.^10,25–29^ Membrane scaling negatively impacts the ED performance by increasing the membrane resistance, decreasing the ion exchange capacity, physically puncturing membranes, and reducing the effective membrane area.^30–33^ Common precipitates formed using hard wastewater are CaCO_3_, Ca­(OH)_2_, gypsum (CaSO_4_·2H_2_O), MgNH_4_PO_4_, and Mg­(OH)_2_, depending on the composition of the wastewater.^32,34,35^ Organic matter further complicates the use of ED in wastewater by promoting or inhibiting scaling nucleation.^32,36,37^

Most investigations characterize the electrodialysis performance using single-salt, synthetic wastewater recipes or treated real wastewater as feedstock to understand the potential for ammonia recovery, prioritizing metrics such as the final product concentration or SEC.^8,9,14^ However, these investigations often overlook understanding the impacts of the wastewater mixture composition on the ED performance. Understanding the interplay between competing ions and molecules is critical to optimizing ED systems for real-world wastewater applications where the wastewater composition and nitrogen concentrations vary widely.^23,38,39^ Such differences in the composition alter the conductivity and selectivity of ions, ultimately impacting electrodialysis energy consumption and concentration factor performances. For example, the permselectivity and membrane potential of PC-SK membranes decrease by 14% and 23%, respectively, by simply changing the electrolyte from ammonium chloride to ammonium bicarbonate.^40^ 5-fold differences in energy consumption and current efficiency for ED ammonium recovery were observed between digestates with composition and pH differences.^9^

To address these discrepancies, here, we compare and investigate ammonium recovery using ED with various feed streams. Due to the complexity of real wastewater, we specifically compare feed streams of similar ammonium concentrations to evaluate the role of the composition. We compare the energy and selectivity of ED systems when ammonia is recovered from mixtures with different inorganic compositions: single-salt (ammonium chloride), simulated wastewater (WW), and real wastewater feed streams. We specifically aim to examine what pretreatment is necessary to obtain a sustained performance. Finally, we discuss the potential product formed from the ED cell and the implications it may have if used as a fertilizer.

## Methods and Materials

### Single- and Multicomponent Wastewater

Analytical-grade reagents and deionized (DI) water were used to prepare the 0.4 M Na_2_SO_4_ electrolyte rinse solution and wastewater mixtures. NH_4_^+^ single-salt wastewater was prepared to mimic the NH_4_^+^–N concentration of wastewater by adding 1,554 mg of NH_4_Cl in 1 L of DI water. Simulated WW was designed to closely replicate the composition of real swine WW found on lagoon surfaces, while simulated WW without hardness (also known as pretreated synthetic WW) excluded Ca^2+^ and Mg^2+^ from synthetic WW. Real wastewater was collected after the sludge dewatering process at the Lubbock Municipal Wastewater Treatment Plant and was processed using an ultrafiltration system (iSpring CU-A4 0.01 μm Ultra-Filtration, iSpring Water Systems) to remove solids and hardness. Details of the ionic compositions for each wastewater type are provided in Tables S1 and S2.^23^

### Electrodialysis for Concentrating Nitrogen Waste

Batch electrodialysis experiments were conducted using a micro ED (PCCell GmbH, Germany) consisting of a Pt/Ir-coated titanium anode, a stainless steel cathode, three cell pairs (6 cm^2^ active area per membrane), and silicone/polypropylene spacers (0.45 mm thickness per spacer). Each cell pair consists of one anion exchange membrane (PC SA, PCCell GmbH, Germany) and one cation exchange membrane (PC-SK, PCCell GmbH, Germany) with spacers between membranes. An end cation exchange membrane (PC MTE, PCCell GmbH, Germany) was placed adjacent to the anode to mitigate transmembrane pressure drops. ED experiments comparing the NH_4_^+^ single-salt solution had diluate batches replaced with a fresh batch once it reached a removal percentage of 60% in terms of conductivity (Thermo Orion Star A215 m with a Thermo Orion 013005MD rugged DuraProbe 4-electrode conductivity cell). Simulated WW without hardness and real WW diluate batches were replaced at the same time intervals as those of the NH_4_^+^ single-salt solution (3.6 and 7.2 h). For staged ED, simulated WW without hardness was concentrated through three consecutive stages of ED. Details of the operating conditions for staged ED are provided in Table S3.

Voltage and energy consumption (BioLogic VMP3 multichannel potentiostat) was recorded as a constant current was applied. Energy consumption (EC) was used to calculate the SEC (kWh/kg-N) defined as the energy consumed to recover a unit mass of NH_4_^+^–N (eq 1)­

$$\mathrm{SEC}=\frac{\mathrm{EC}}{0.7766\cdot \Delta C_{{{\mathrm{NH}}_{4}}^{+}}\cdot V\left(t\right)}$$ 1

where *t* is the ED operation duration (min), 0.7766 is a unit conversion of kg-NH_4_^+^ to kg-N, EC is the cumulative energy consumption over time *t*, Δ*C*_NH_4_^+^_ is the change in the NH_4_^+^ concentration (mg/L) over time *t*, and *V*(*t*) is the volume (L) of the concentrate at time *t*. The pumping energy is assumed to be negligible when scaled up and is not included in the SEC calculation.^18^ Ion concentrations of diluate and concentrate streams were measured using cation and anion ion chromatography (Dionex Aquion, IonPac CS12A column, and IonPac AS22 column). Concentration measurements were used to calculate concentration factors, transport numbers, normalized transport numbers, selectivity, and theoretical ammonium concentration ignoring osmosis effects (eqs 2, 3, 4, 5 and 6):^41^

2 $$c\mathrm{oncentration}\;\mathrm{factor}\;\left(C/C_{0}\right)=\frac{C_{i}\left(t\right)}{C_{i}\left(0\right)}$$

3 $$t_{i}=\frac{\mathrm{zF}\Delta n_{i}}{\mathrm{It}}$$

4 $$t_{\mathrm{norm},i}=\frac{t_{i}}{C_{i}\left(0\right)/\sum_{i}^{\mathrm{ions}}C_{i}\left(0\right)}$$

$$s\mathrm{electivity}=\frac{t_{a}/t_{b}}{\overset{¯}{C_{a}}/\overset{¯}{C_{b}}}$$ 5

$$t\mathrm{heoretical}\;{{\mathrm{NH}}_{4}}^{+}\;\mathrm{concentration}\;\left(g/L\right)=C_{{{\mathrm{NH}}_{4}}^{+}}\;\left(t\right)\times \frac{V\left(t\right)}{V\left(0\right)}$$ 6

where *C*_i_(*t*) and *C*_i_(0) represent the concentrations of ion i at time *t* and 0; *V*(*t*) and *V*(0) are the concentrate volumes at time *t* and 0; *t*_i_ is the transport number; *t*_norm,i_ is the transport number normalized by the initial ratio of ion i; *z* is the valency of i; *F* is Faraday’s constant; *I* is the total current applied; Δ*n*_i_ is the change in the number of moles of ion i of the concentrate stream; and $\overset{®}{C_{i}\;}$ is the average concentration of i over time *t*. Although the normalized transport number and selectivity both address the transport of ions through ion exchange membranes, selectivity is used to describe the intrinsic selective transport induced by the membrane, while the normalized transport number includes changes in transport or concentration due to nonmembrane characteristics such as membrane scaling. The theoretical NH_4_^+^ concentration represents the hypothetical ammonium concentration unaffected by osmotic water transport, assuming that osmosis does not significantly affect the flux of NH_4_^+^ ions and volume changes are due to osmotic water flux.

Dimensionless time was introduced to normalize time against residence time, as shown below

7 $$t^{{\;}^{*}}=\frac{t}{\tau }$$

where *t* ^*^ is the dimensionless time and τ is the residence time.

## Results and Discussion

### Ammonia Recovery with Synthetic Wastewater

When ammonia is concentrated by batch electrodialysis with a simulated WW mixture, the cations concentrated from the wastewater feed include NH_4_^+^, K^+^, Na^+^, and Mg^2+^ (Figure 1a). However, the cell reached high resistances of 350 Ω (20 V) before observing the decrease in diluate conductivity by 60% (Figure S2b). This abnormally high resistance increase was attributed to the accumulation of calcium scalants formed in the electrodialysis cell. Unlike NH_4_^+^, K^+^, Na^+^, and Mg^2+^, the Ca^2+^ concentration remained constant (approximately 100 mg/L) in the concentrate, and the normalized transport number was less than 0.1, indicating that calcium was not transferred across the membrane due to electromigration. Less calcium transport was anticipated because calcium is known to readily precipitate in the presence of phosphate.^28^ Therefore, the change in the concentration of calcium ions in solution is low because calcium ultimately separates as a solid. From a membrane and system perspective, this precipitation can cause detrimental scaling, which increases energy consumption through increased system resistance (Figure S2b).

**Figure 1:**
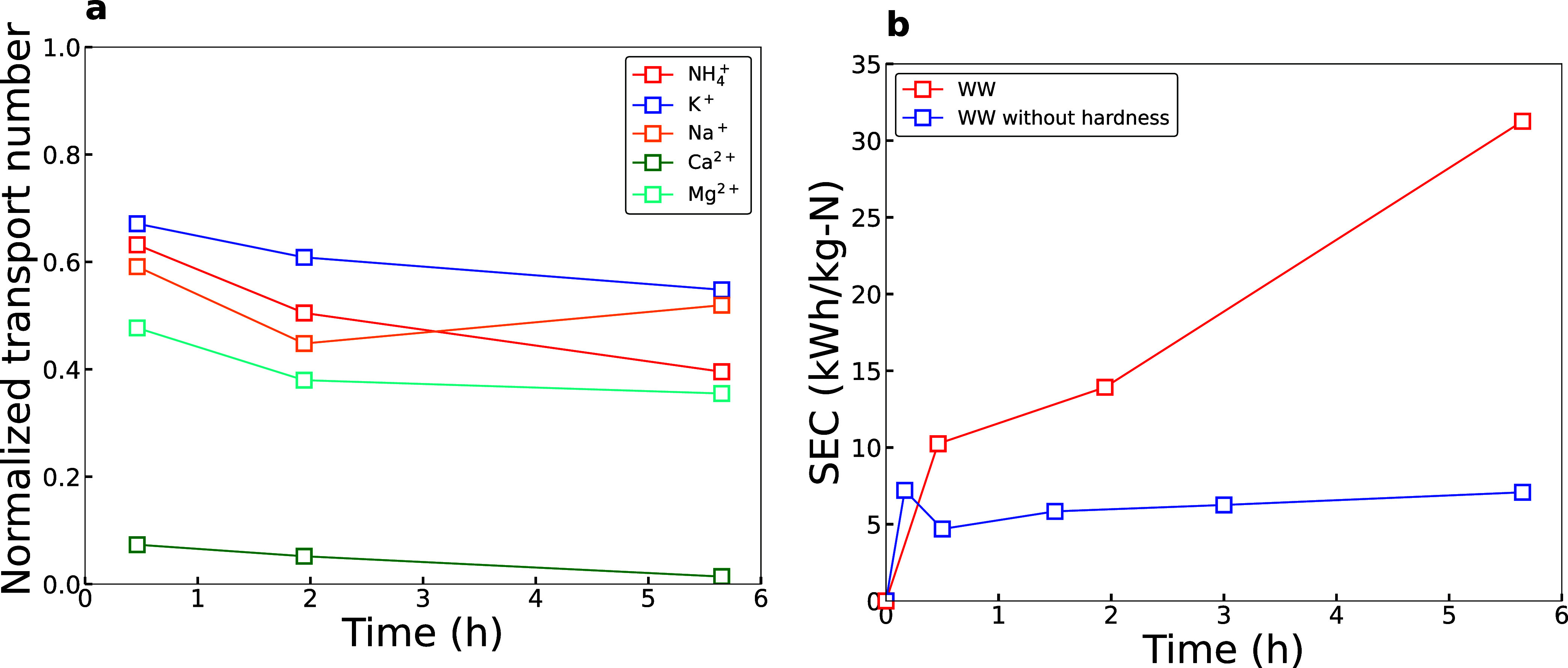
(a) Normalized transport numbers of ions in WW with hardness and (b) SEC comparison of WW with hardness vs WW without hardness.

Calcium precipitates were detected on the membranes (Figure S4). X-ray diffraction identified the precipitate as carbonate apatite (Ca_10_(PO_4_)_6_CO_3_), a common precipitate formed from wastewater containing urea or other carbonate sources (Figure S5).^37^ The resulting SEC was 31.26 kWh/kg-N for WW without pretreatment (e.g., with hardness present). Next, we examined simulated WW where the hardness was removed. This could be achieved in practice using hardness-removing technologies such as electrocoagulation, ultrafiltration, chemical precipitation, etc. Removing the hardness decreased the SEC by 4-fold to 7.08 kWh/kg-N, underlining the importance of removing divalent ions like Mg^2+^ and Ca^2+^ from wastewater solutions (Figure 1b).^42^

NH_4_^+^, K^+^, and Na^+^ displayed similar normalized transport numbers of 0.5–0.6. This highlights a selectivity challenge that exists with electrodialysis even after WW pretreatment. Since the membranes are selective to all cations, we see that all monovalent ions from WW mixtures are transported equally across the membrane and will equally concentrate NH_4_^+^, K^+^, and Na^+^ (Figure S2a). However, potassium and sodium are beneficial nutrients, and thus the recovery of potassium and sodium can be advantageous for the production of fertilizers from wastewater.^43,44^ Potassium is one of the three NPK macronutrients (nitrogen–phosphorus–potassium) crucial for plant growth. It facilitates water movement and nutrient transport throughout the plant. Sodium is viewed as a functional nutrient by promoting maximal biomass growth, being an osmoticium for cell enlargement, and replacing potassium for long-distance nutrient transport. However, plant species, soil characteristics, and environmental conditions influence tolerance; sodium concentrations above 0.03–0.2 M can degrade soil permeability and must be managed with caution.^43,45,46^ Specifically, high sodium uptake by plants could decrease biomass accumulation, deform plant sensory functions, and alter cellular biochemistry.^47–51^ To reduce this risk associated with excess sodium, there is motivation for the fabrication of selective cation exchange membranes toward NH_4_^+^ and K^+^ relative to Na^+^.^52,53^ Integration of hollow fiber membrane contactors downstream of ED also promotes the concentration and selective separation of NH_4_^+^.^8^

### Ammonia Recovery with Simulated and Real Wastewater

To evaluate the concentration factor of ED, we establish our control ammonia solution as a single-salt solution (nonmixture) and compare it with the simulated and real WW mixtures (Figures 2 and S7). With 10 mA/cm^2^ applied for three diluate batches over ten hours, the maximum CF achieved was ∼9× (5170 mg/L) for the single-salt nonmixture. Unlike single-salt nonmixtures, simulated WW with pretreatment has additional cations in the mixture to carry the current. The CF of WW without Ca^2+^ and Mg^2+^ exceeded that of the single-salt mixture after 9 h and reached a maximum concentration of ∼10× (5740 mg/L). Finally, we found that the maximum CF of ammonium in the ED cell using real WW (without pretreatment) reached ∼7.5× (5690 mg/L).

**Figure 2:**
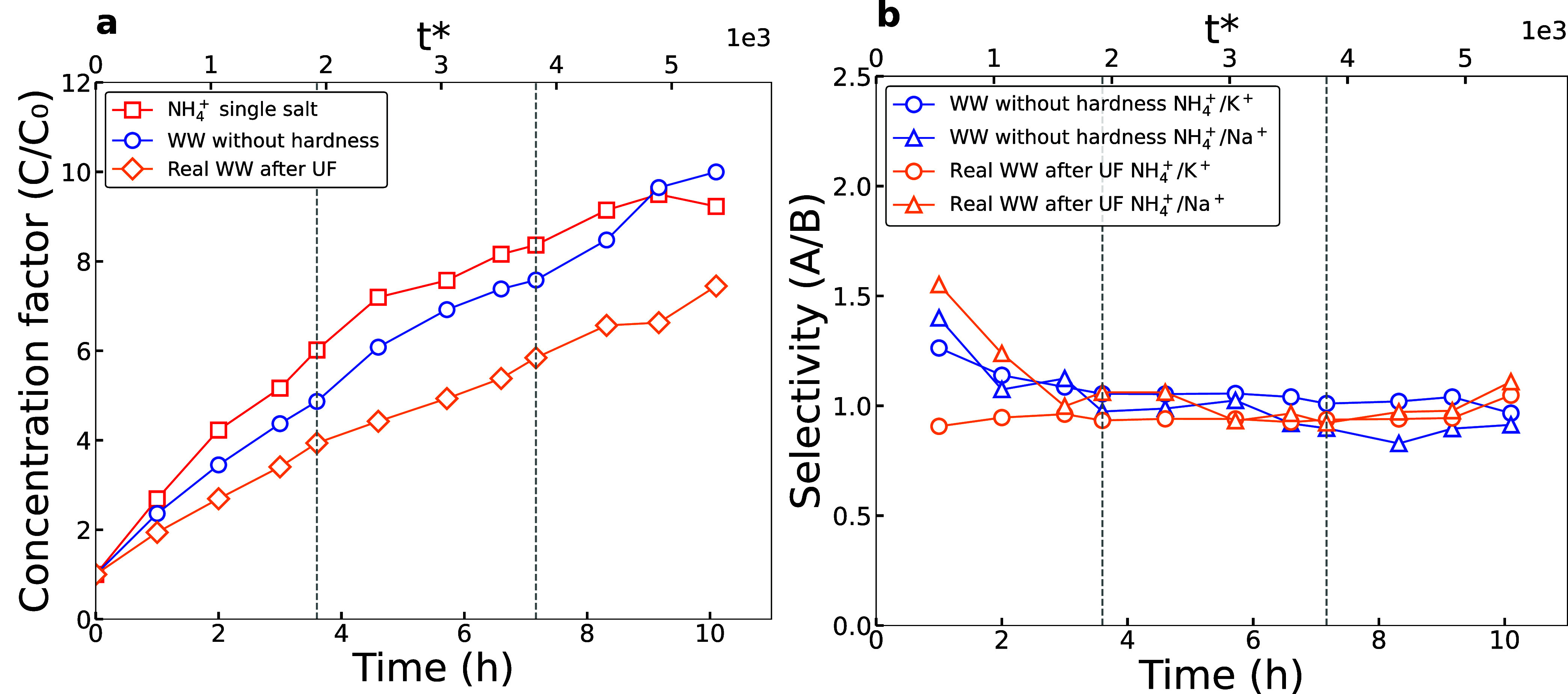
(a) Concentration factor trend against time for the single-salt solution (red), WW without hardness (blue), and real WW (yellow). (b) Selectivity of NH_4_^+^ against K^+^ and Na^+^ shown for WW without hardness (blue) and real WW (yellow). Black dotted lines represent the replenishment of the diluate batch.

The maximum ammonium concentration of the single-salt solution (5170 mg/L) was lower than that of the wastewater mixtures due to the increased flux of water. The osmotic flow of water increased the concentrated volume by 60% for the single-salt solution, 10% for WW without pretreatment, and 17% for real WW without pretreatment. This difference could stem from different factors such as the total solute concentration, composition, and pH, which affects the Donnan potential, electro-osmosis, and bulk osmosis forces.^54–56^ However, wastewater mixtures are very complex with a myriad of variables, and an in-depth study of osmosis forces requires vigorous control experiments that will be further investigated in the following studies. Significant transport of osmotic water to the concentrate is disadvantageous for ED because osmosis impedes the rate of concentration. For example, the theoretical concentration factor for a single salt (Figure S7) shows that a concentration of 8.2 g/L (a concentration factor of ∼8×) could have been achieved with minimal osmotic water flux.

The selectivities of NH_4_^+^ versus K^+^ and Na^+^ (Figure 2b) were all close to unity, showing no preference for a specific counterion. This was expected because the transport behavior of NH_4_^+^, K^+^, and Na^+^ is often very similar across sulfonic cation exchange membranes.^57^ Thus, ED using mixtures of NH_4_^+^, K^+^, and Na^+^ will result in product streams with all three counterions present.

To obtain fertilizer-grade nitrogen nutrient levels, synthetic WW without hardness was concentrated using staged ED (Figures 3 and S10). For stage 1 ED, 6.88 g-NH_4_^+^/L (0.66 N wt %, CF of ∼13) with a SEC of 15.18 kWh/kg-N was reached (Figure 3a). K^+^ reached 7.50 g/L (CF of ∼9) and Na^+^ reached 2.43 g/L (CF of ∼12). Stage 2 and stage 3 ED used a concentrated version of the simulated WW with pretreatment based on the NH_4_^+^ concentration of the previous stage. Stage 2 reached 16.03 g-NH_4_^+^/L (1.52 N wt %, CF of ∼2.5) with a SEC of 18.08 kWh/kg-N, and stage 3 reached 25.31 g-NH_4_^+^/L (2.42 N wt %, CF of ∼1.6) with a SEC of 17.25 kWh/kg-N (Figure 3b,c). The cumulative concentration factor for the three stages was ∼49.5×. The nitrogen weight percentage after stage 3 ED was very similar to results from concentrating single-salt NH_4_Cl through a staged ED operation (2.15 N wt %), though requiring 3–10 times the SEC.^8^ The higher SEC for mixtures can again be attributed to the fact that all current applied in a single-salt solution is used to transport NH_4_^+^, while in a mixture, a portion of the current is used to transport other cations such as Na^+^ and K^+^. To reach a fertilizer-grade nitrogen concentration level of 10 N wt % (∼200× concentration factor), the effluent mixture could be connected with a hollow fiber membrane contactor (HFMC).^8^

**Figure 3:**
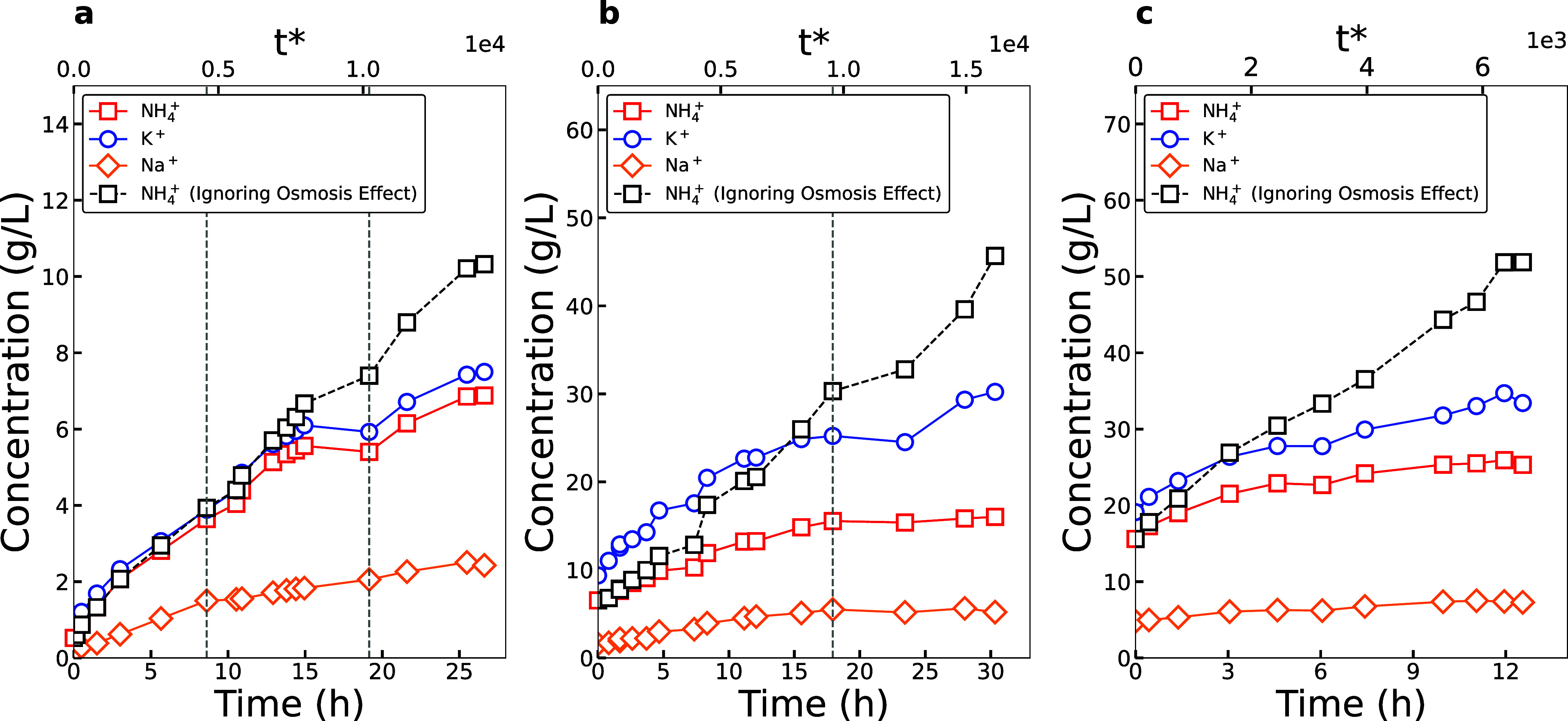
Ionic concentration change over time for (a) stage 1 ED, (b) stage 2 ED, and (c) stage 3 ED. Concentration change for NH_4_^+^ ignoring osmosis (black) is also shown. Black dotted lines represent the replenishment of the diluate batch.

Osmotic water flux occurred throughout staged ED and prevented further ion concentration, significantly decreasing the final nitrogen content available (Figure 3). Without osmosis, stage 1 would have reached 10.3 g-NH_4_^+^/L (0.99 N wt %, CF of ∼20) with 10.12 kWh/kg-N, stage 2 would have reached 45.7 g-NH_4_^+^/L (4.36 N wt %, CF of ∼7) with 6.30 kWh/kg-N, and stage 3 would have reached 51.9 g-NH_4_^+^/L (4.95 N wt %, CF of ∼3.3) with 8.43 kWh/kg-N. Combined, 87.0 g-NH_4_^+^/L (8.32 N wt %, ∼166× concentration factor) could have been reached. This 3.4× difference in concentration with and without osmosis highlights the lost opportunity in product recovery, which can be mitigated through reinforced ion exchange membranes against water permeance. While complete suppression of osmotic water flux remains challenging, strategies such as membrane polymer backbone modification,^58^ surface coating application,^59^ and the development of composite membranes^60,61^ have been explored to regulate water transport. Even a 50% decrease in osmotic water flux could increase the cumulative concentration factor to 105, yielding a 5.24 N wt % product. The SEC of the system also decreases by 2–3× without osmosis. This reduction in the SEC resulting from minimized osmotic water transport could significantly enhance the competitiveness of ED relative to other ammonia recovery technologies with comparable energy consumption, such as ammonia stripping (1.2–4.0 kWh/kg-N),^38,62^ reverse osmosis (3.7–4.7 kWh/kg-N),^38,63,64^ electrochemical processes with column stripping and absorption (6.5–13 kWh/kg-N),^8,21,65^ and capacitive deionization (20.6–35.4 kWh/kg-N).^66–68^ This advantage is particularly noteworthy, given that ED does not require chemical additives, in contrast to ammonia stripping and electrochemical processes with stripping, where chemical costs constitute a major portion of the overall operational expenditure.^24,69^ Therefore, reinforced ion exchange membranes would be beneficial, especially for the latter stages where osmosis is more pronounced, for a higher performance of ED ammonia recovery.

## Conclusions

Here, we compare and demonstrate the recovery of ammonia from wastewater mixtures. ED operated without pretreatment (e.g., hardness removal) resulted in the membrane scaling of Ca_10_(PO_4_)_6_CO_3_. Removing the hardness (Mg^2+^ and Ca^2+^) from simulated WW decreased the SEC from 31.26 to 7.08 kWh/kg-N, highlighting the need for hardness pretreatment methods prior to ED. The single-salt solution, the simulated WW with pretreatment, and the real WW achieved different NH_4_^+^ concentrations of 5.2 g/L, 5.7 g/L, and 5.7 g/L. The selectivity of NH_4_^+^ against K^+^ and Na^+^ and the flux of osmotic water at high-concentration gradients between the diluate and the concentrate affected the concentration rate and the final concentration. For the mixtures, K^+^ and Na^+^ were present in the final product and were not selectively separated. Simulated WW with pretreatment in staged ED resulted in 25.3 g-NH_4_^+^/L (2.4 N wt % concentrate, ∼49.5×) with SECs of 15.2–18.1 kWh/kg-N. Without osmosis, 87.0 g-NH_4_^+^/L (8.32 N wt % concentrate, ∼166×) can be reached with SECs of 6.3–10.1 kWh/kg-N.

## Supplementary Material



## Notes

Literature review for membrane scaling on ED; single-salt solution, simulated WW, and real WW composition data; operating conditions for ED; photo of ED; normalized transport number of ions in simulated WW after pretreatment; X-ray diffraction spectrum for the scalant; and the concentration trend, CF trend, voltage, and energy consumption data for different wastewater mixtures and stages 1–3 for staged ED (PDF)
