# Supplementary material for: Concentrating Ammonia from Wastewater with Electrodialysis
Source: ACS ES T Water. 2025 Aug 19;5(9):5720–7. doi: 10.1021/acsestwater.5c00721 (PMC12439302; doi:10.1021/acsestwater.5c00721)
Supplement: Supplementary file 1 [file ew5c00721_si_001.pdf]

Supporting Information: Concentrating Ammonia from Wastewater with Electrodialysis

Hyuck Joo Choi,[†] Mohammed Tahmid,[†] Spandan Mondal,[†] and Marta C. Hatzell*[,‡,†]

†*School of Chemical and Biomolecular Engineering, Georgia Institute of Technology, 311 Ferst Drive NW, Atlanta, GA, 30332, United States*

‡*George W. Woodruff School of Mechanical Engineering, Georgia Institute of Technology, 770 Ferst Drive NW, Atlanta, GA, 30332, United States*

E-mail: marta.hatzell@me.gatech.edu

Phone: +123 (0)123 4445556. Fax: +123 (0)123 4445557

Tables

Table S1: Single salt, simulated WW, and simulated WW without hardness recipe in 1L DI water

**Component**	**Single Salt WW (mg)**	**Simulated WW (mg)**	**Simulated WW without Ca^{2+} and Mg^{2+} (mg)**
NH_4Cl	1554	1554	1554
$Na_2HPO_4{\cdot}H_2O$	-	655	655
Urea	-	168	168
NaCl	-	40	40
Na_2SO_4	-	165	165
KCl	-	785	785
$CaCl_2$	-	286	-
$MgCl_2$	-	215	-

Table S2: Real wastewater composition after ultrafiltration in 1L DI water

**Component**	**Real WW after UF (mg)**
NH_4^+	764
K^+	200
Na^+	262
Cl^-	227
PO_4^{3-}-P	211
SO_4^{2-}	37
Dissolved Organic Carbon (DOC)	900 - 2000

Table S3: Operating conditions for ED

**Condition**	**ED Stage 1**	**ED Stage 2**	**ED Stage 3**
Flow rate (L/h)	1.44	1.44	1.44
Current density (ED) (mA/cm^2)	10	40	67
Number of cell pairs (ED) (AEM+CEM)	3	3	3
Volume of diluate (ED) (mL)	1100	1100	1100
Volume of concentrate (ED) (mL)	100	100	100
Number of diluate batches (ED)	3	2	1

Table S4: Comparison of scalant formed for studies using electrodialysis to recover and concentrate ammonia from wastewater

**Electrochemical cell configuration**	**CEM**	**AEM**	**Scalant**	**Current Density (mA/cm^2)**	**Membrane Area (cm^2)**	**Calcium Feed (mg/L)**	**Magnesium Feed (mg/L)**	**Reference**
CCAA	Selemion CMV, CR67	Selemion AMV, AR908	$CaCO_3$, $MgNH_4PO_4$	5.1-16.9	4	104, 140	9.7, 80	[1]
Standard ED	CJMC-4	CJMA-4	$CaCO_3$, $Ca(OH)_2$, $Mg(OH)_2$	14	189	419	1327	[2]
Standard ED	MK-40, MK-40 modified	MA-41	$CaCO_3$, $Ca(OH)_2$, $Mg(OH)_2$	4-18	4	753.6	291.8	[3]
ED + RO	PC-cell CEM	PC-cell AEM	$CaSO_4 \cdot 2H_2O$	40	64	723	N/A	[4]
Standard ED	Fujifilm CEM-Type 12	Fujifilm AEM-Type 12	$CaSO_4$, $Ca(OH)_2$	0.95-2.02	84	500-2000	N/A	[5]
Standard ED	Selemion CMV, MK-40	MA-40	$CaSO_4 \cdot 2H_2O$	6-12	43.8	4008	N/A	[6]
Standard ED	General Electric CR67	General Electric AR204-SRZA	(only elemental characterization) Ca, Mg, C, P, O, organics	29.8	168	4.37	42.1	[7]
Standard ED	Neosepta CMX-S	Neosepta AMX-SB	$Ca(OH)_2$	40	10	145-578	N/A	[8]
Standard ED	Neosepta CMX-S	Neosepta AMX-SB	$CaCO_3$, $Ca(OH)_2$, $Mg(OH)_2$	40	10	289	14.4-115	[9]

ED: Electrodialysis with alternating CEM and AEM, CEM (C): Cation Exchange Membrane, AEM (A): Anion Exchange Membrane, RO: Reverse Osmosis, N/A: Not Available

Table S5: Comparison of performance metrics of electrochemical cell studies to recover and concentrate ammonia from wastewater

**Method**	**Electrochemical cell configuration**	**Feed NH_4^+ concentration (mg/L)**	**Current Density (mA/cm^2)**	**Concentration Factor for NH_4^+**	**Energy Consumption (kWh/kg-N)**	**Reference**
This study	3 cell pairs	524 (simulated WW without hardness)	10-67	49.5	15.2-18.1	
ED + HFMC	ED with 5 cell pairs	524	0.75-6	200	1.89-6.14	[10]
Electrochemical Stripping	Single CEM	7490	10	0.3	1010	[11]
BMED + BMED	5 cells for each BMED	5400	16.7	2.96	N/A	[12]
BMED	10 cell pairs	3410	75	4.03	3.81	[13]
LLMC + ED	ED with 5 cell pairs	1700-4000	Variable (constant voltage = 7V)	1.5-2.2	0.9-8.9	[14]
BMED + GPM	ED with 1 cell pair	3360-3772	20	2.5	25.8	[15]
ED	30 cells	835	2	8.5	4.95	[16]
ED	10 cells	1500	dynamic (change according to conductivity)	6.7	N/A	[17]

ED: Electrodialysis, BMED: Bipolar Membrane Electrodialysis, LLMC: Liquid-Liquid Membrane Contactor, GPM: Gas Permeable Membrane, HFMC:, Hollow Fiber Membrane Contactor, AEM: Anion Exchange Membrane, CEM: Cation Exchange Membrane, BPM: Bipolar Membrane, N/A: Not Available

Figures

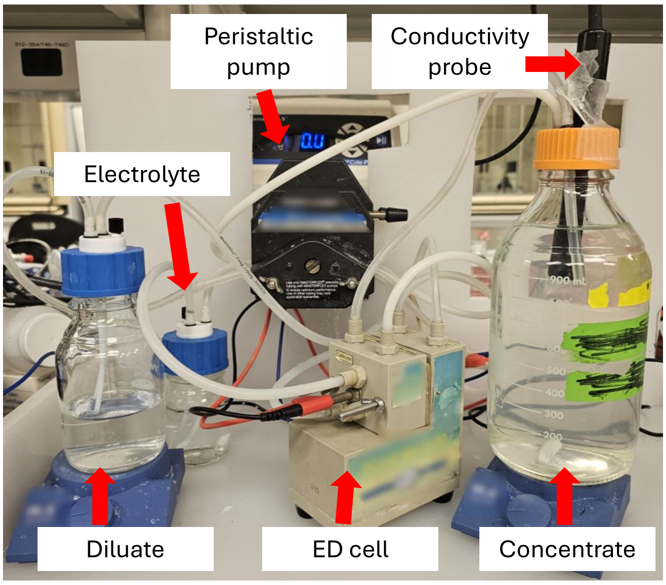

Figure S1: Picture of ED system setup

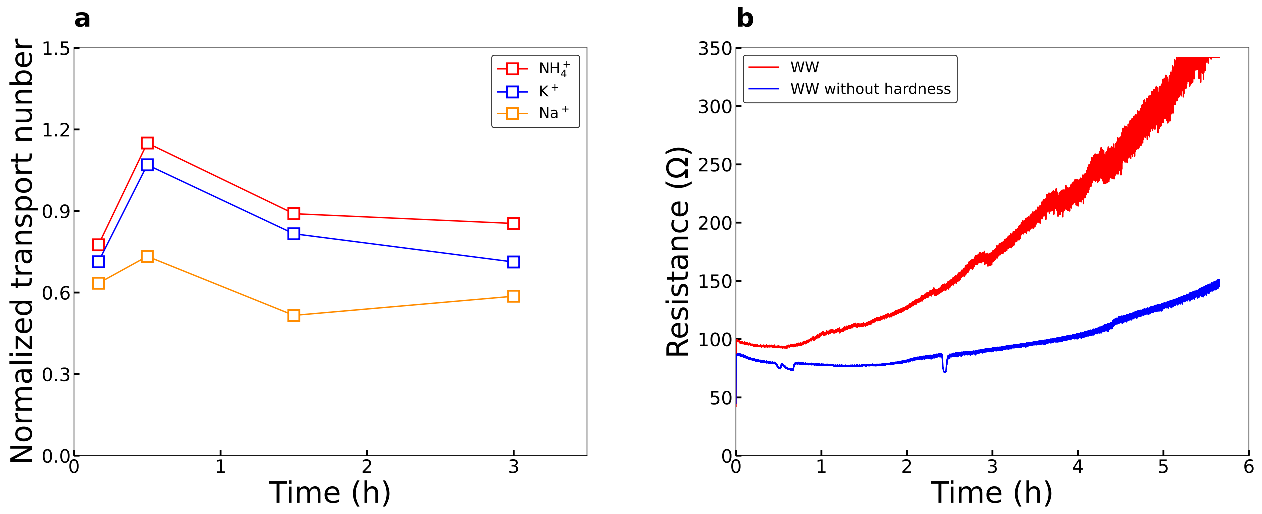

Figure S2: (a) Normalized transport number of ions in WW with preteatment(b) resistance comparison of WW with and without hardness

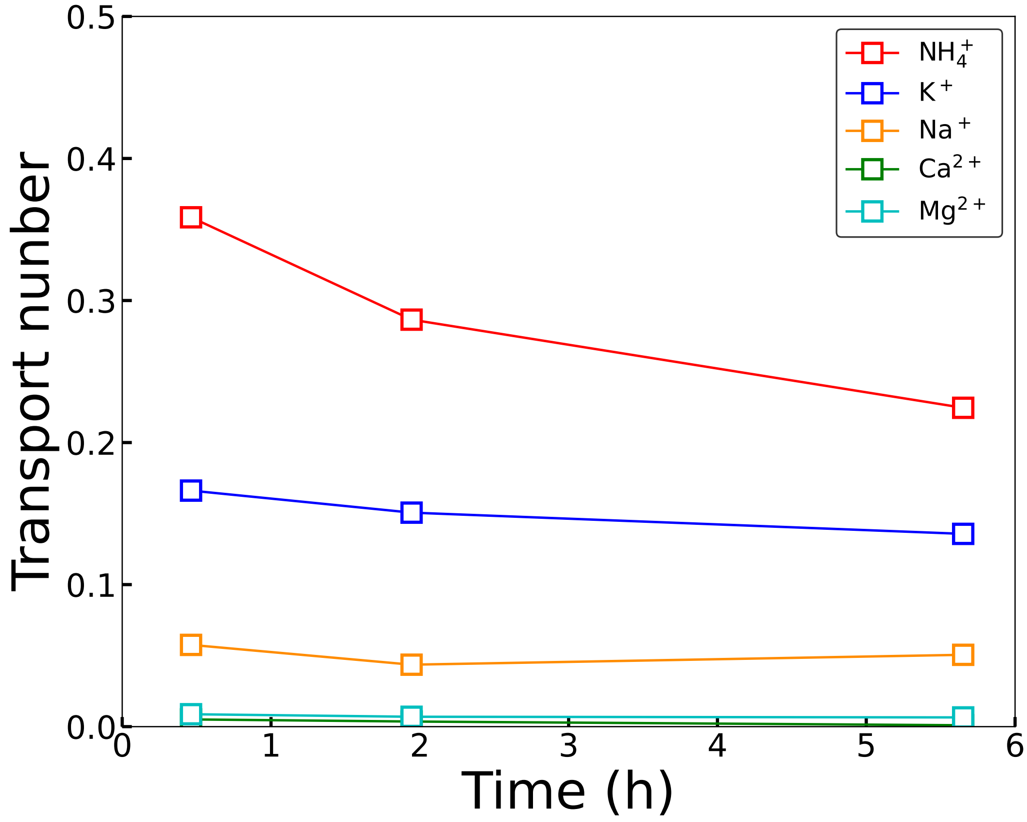

Figure S3: Transport number of ions in WW with hardness

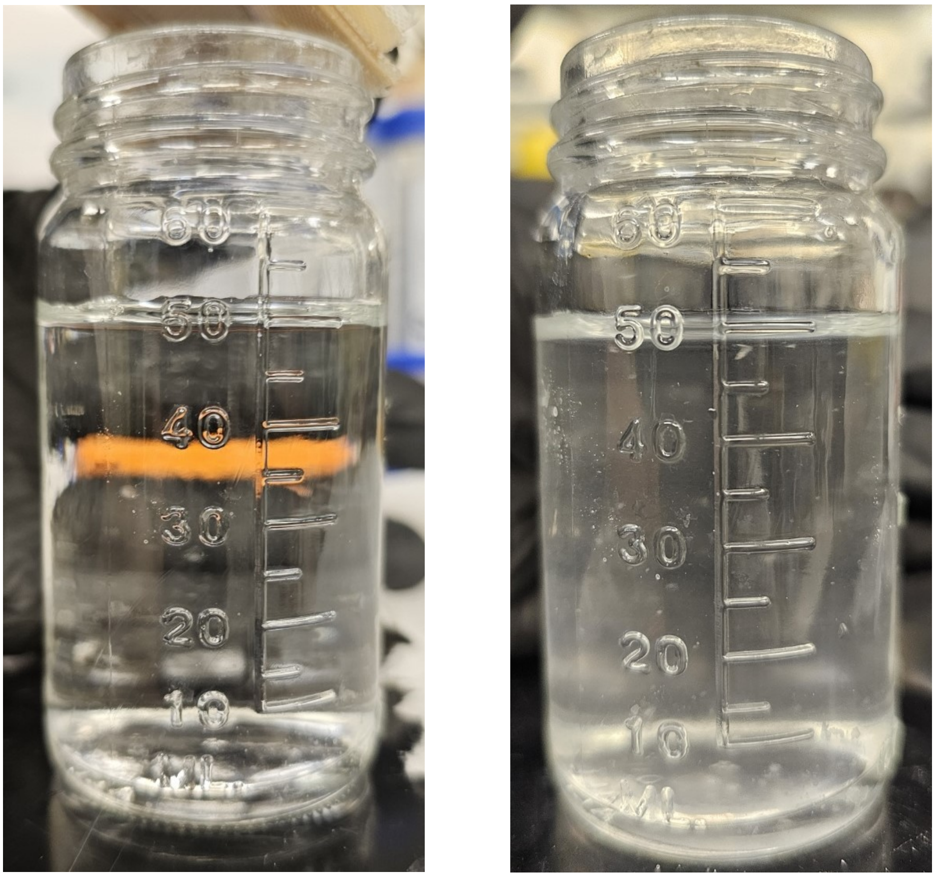

Figure S4: Pictures of DI water after immersing membranes for 1 hour before ED operation (left) and after ED (right) operation.

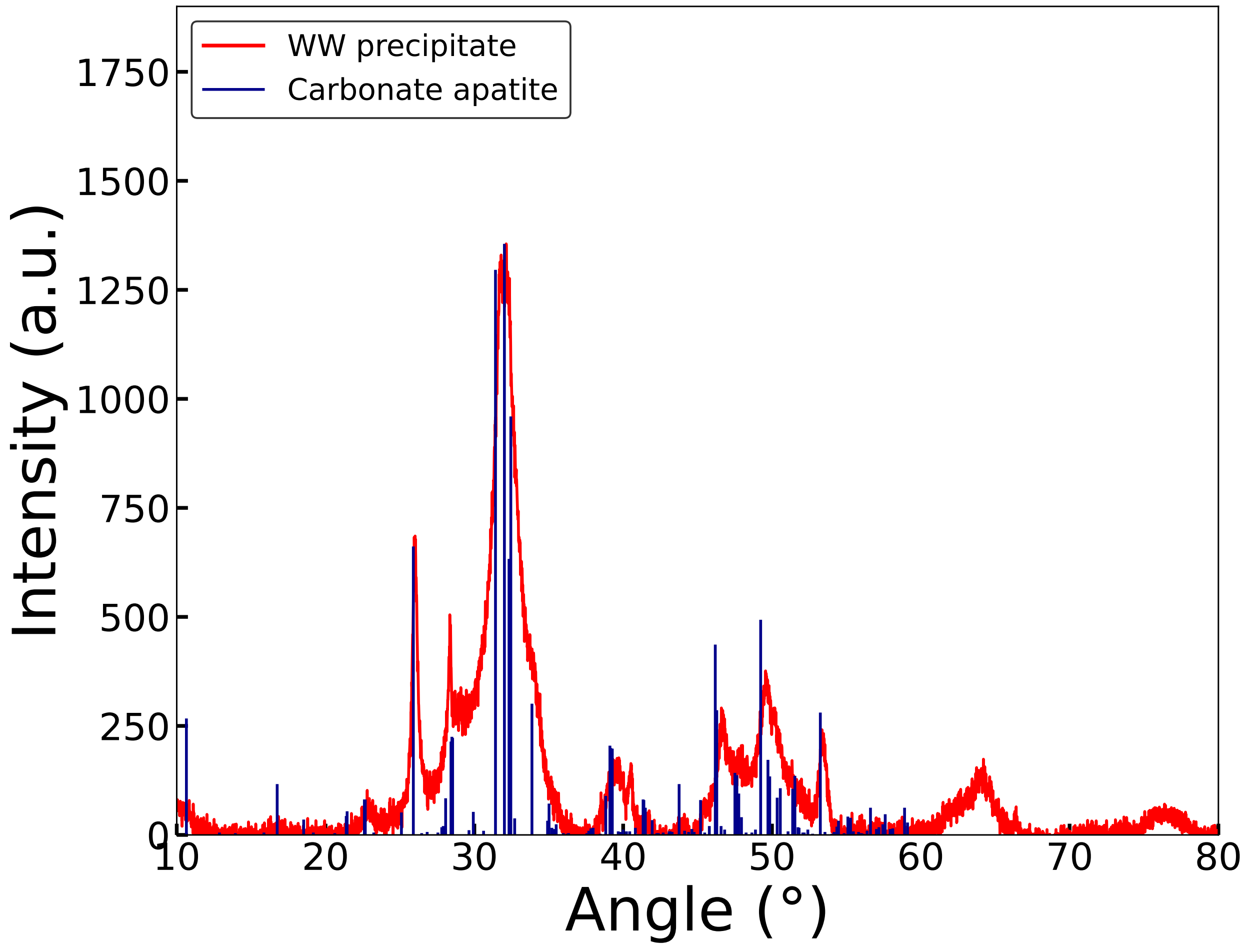

Figure S5: X-ray diffraction pattern of precipitate formed after electrodialysis using simulated WW

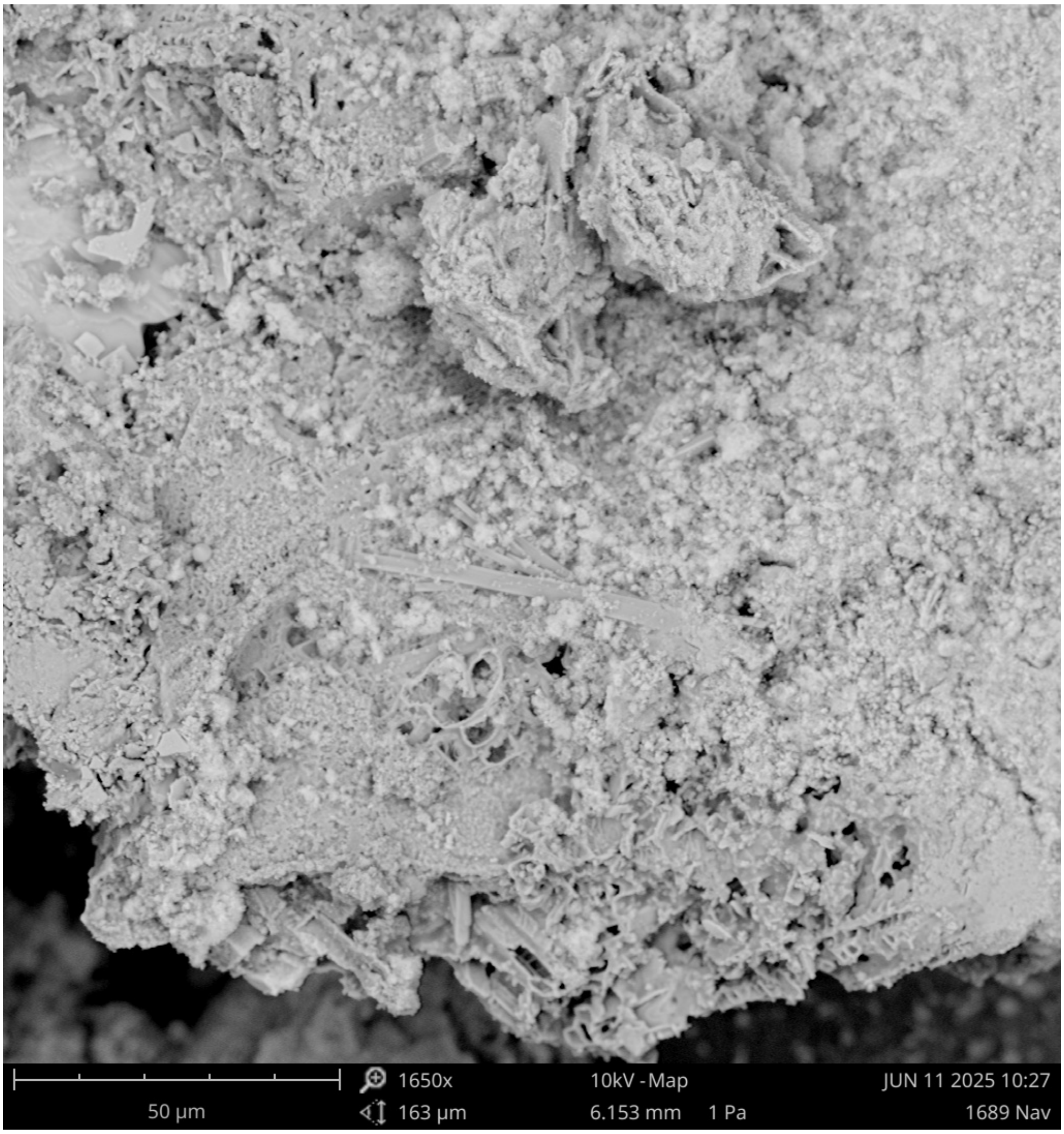

Figure S6: SEM of precipitate formed after electrodialysis using simulated WW

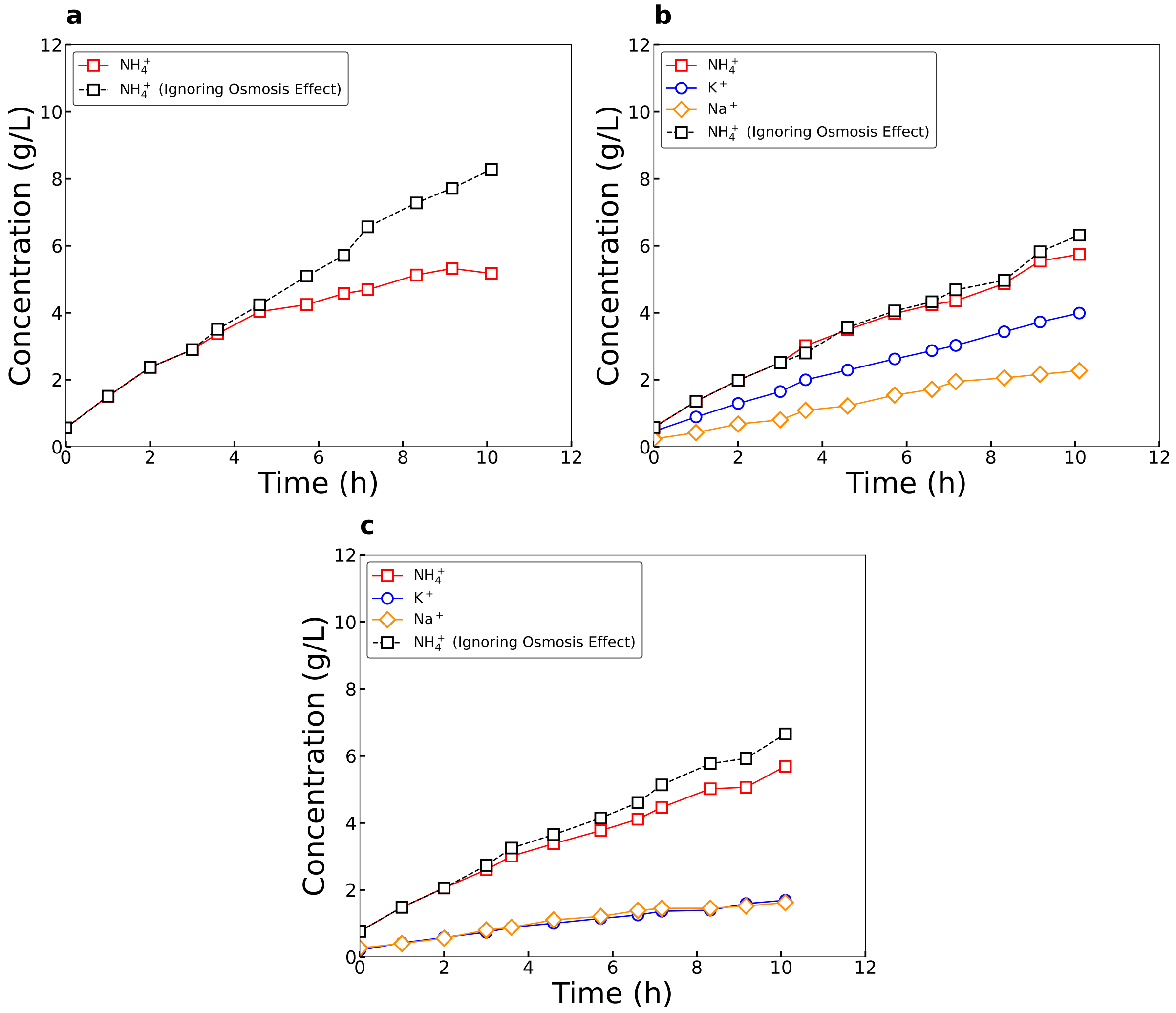

Figure S7: Ion concentration against time for (a) single salt ammonium feed, (b) simulated WW with pretreatment feed, and (c) real WW without pretreatment using single stage ED

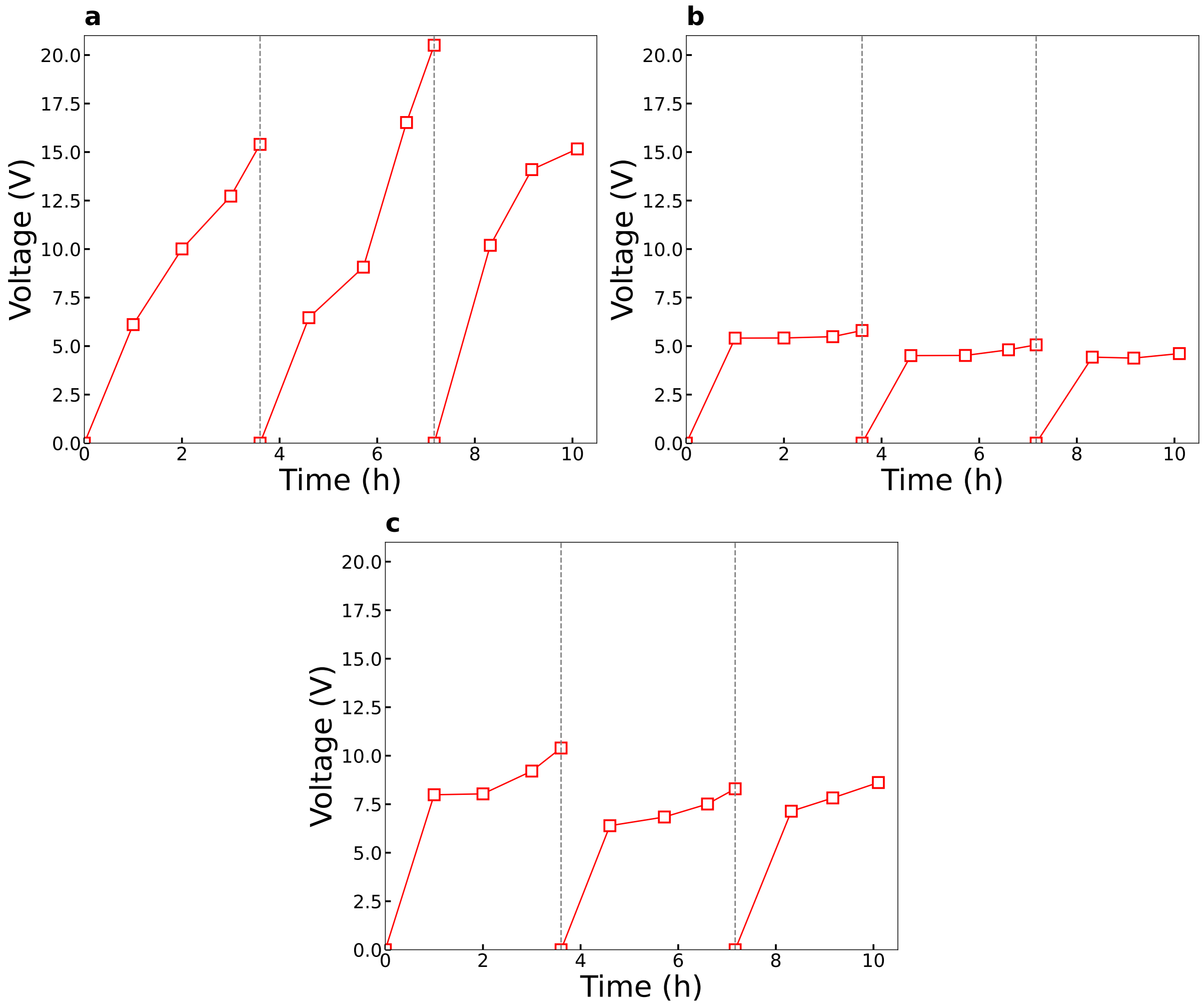

Figure S8: Voltage data for ED operation using (a) NH_4^+ single salt, (b) simulated WW without hardness, and (c) real WW using single stage ED.

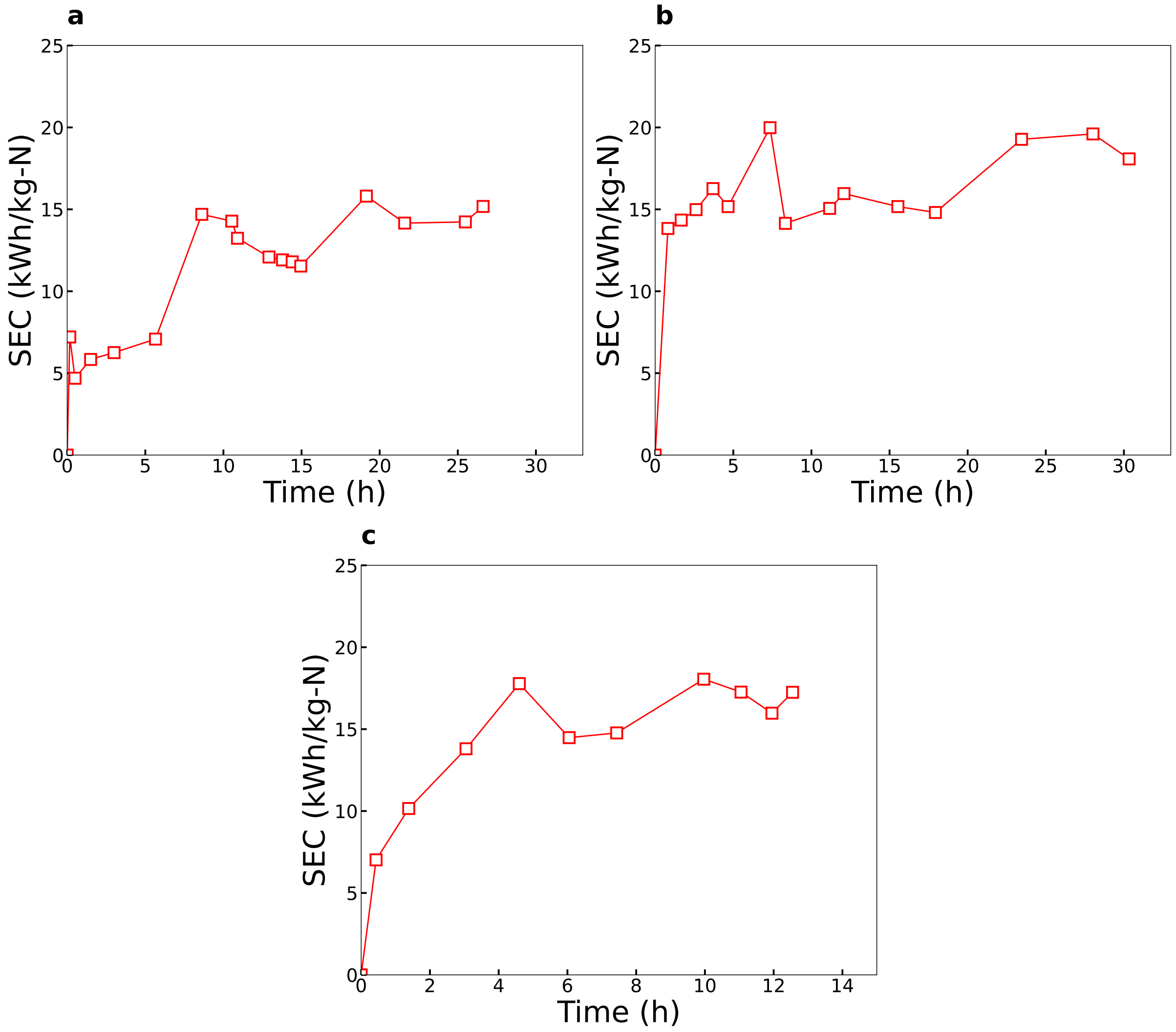

Figure S9: SEC trend against time shown for (a) stage 1, (b) stage 2, and (c) stage 3 ED using simulated WW without hardness

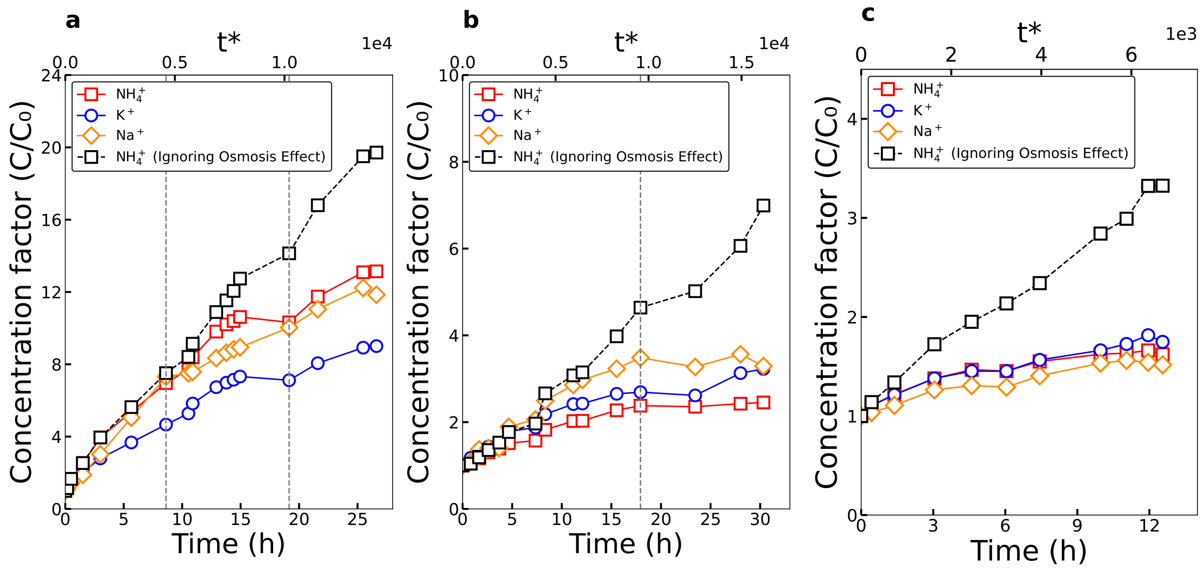

Figure 10: Concentration factor over time for (a) stage 1 ED, (b) stage 2 ED, and (c) stage 3 ED. Concentration factor change for NH_4^+ ignoring osmosis (black) is also shown. Black dotted lines represent replenishment of diluate batch.

References

(1) Guo, H.; Kim, Y. Membrane Scaling in Electrodialysis Fed with High-Strength Wastewater. *Environmental Engineering Science* **2021**, *38*, 832–840, Publisher: Mary Ann Liebert, Inc., publishers.

(2) Liu, H.; She, Q. Scaling-Enhanced Scaling during Electrodialysis Desalination. *ACS ES&T Engineering* **2024**, *4*, 1063–1072, Publisher: American Chemical Society.

(3) Andreeva, M. A.; Gil, V. V.; Pismenskaya, N. D.; Dammak, L.; Kononenko, N. A.; Larchet, C.; Grande, D.; Nikonenko, V. V. Mitigation of membrane scaling in electrodialysis by electroconvection enhancement, pH adjustment and pulsed electric field application. *Journal of Membrane Science* **2018**, *549*, 129–140.

(4) Yao, Y.; Zhang, X.; Wang, R.; Lin, S.; Tong, T. Mineral scaling and organic fouling in electrodialytic crystallization. *Journal of Membrane Science* **2024**, *707*, 123024.

(5) Gou, M.; Zhao, Y.; Li, B.; Li, Y.; Li, Z.; Wang, M. Scaling behavior of cation exchange membrane induced by Ca2+ during the electrodialysis for enriching lithium-containing solutions. *Journal of Membrane Science* **2025**, *713*, 123283.

(6) Asraf-Snir, M.; Gilron, J.; Oren, Y. Scaling of cation exchange membranes by gypsum during Donnan exchange and electrodialysis. *Journal of Membrane Science* **2018**, *567*, 28–38.

(7) Thompson Brewster, E.; Ward, A. J.; Mehta, C. M.; Radjenovic, J.; Batstone, D. J. Predicting scale formation during electrodialytic nutrient recovery. *Water Research* **2017**, *110*, 202–210.

(8) Bazinet, L.; Araya-Farias, M. Effect of calcium and carbonate concentrations on cationic membrane fouling during electrodialysis. *Journal of Colloid and Interface Science* **2005**, *281*, 188–196.

(9) Casademont, C.; Pourcelly, G.; Bazinet, L. Effect of magnesium/calcium ratio in so-

lutions subjected to electrodialysis: Characterization of cation-exchange membrane fouling. *Journal of Colloid and Interface Science* **2007**, *315*, 544–554.

(10) Tahmid, M.; Joo Choi, H.; Ganapavarapu, S. T.; Scott, J.; Hatzell, M. C. Concentrating Nitrogen Waste with Electrodialysis for Fertilizer Production. *Environmental Science & Technology Letters* **2024**, *11*, 1413–1419, Publisher: American Chemical Society (ACS).

(11) Tarpeh, W. A.; Barazesh, J. M.; Cath, T. Y.; Nelson, K. L. Electrochemical Stripping to Recover Nitrogen from Source-Separated Urine. *Environmental Science & Technology* **2018**, *52*, 1453–1460, Publisher: American Chemical Society.

(12) Shi, L.; Xiao, L.; Hu, Z.; Zhan, X. Nutrient recovery from animal manure using bipolar membrane electrodialysis: Study on product purity and energy efficiency. *Water Cycle* **2020**, *1*, 54–62.

(13) Shi, L.; Hu, Y.; Xie, S.; Wu, G.; Hu, Z.; Zhan, X. Recovery of nutrients and volatile fatty acids from pig manure hydrolysate using two-stage bipolar membrane electrodialysis. *Chemical Engineering Journal* **2018**, *334*, 134–142.

(14) Vecino, X.; Reig, M.; Gibert, O.; Valderrama, C.; Cortina, J. L. Integration of liquid-liquid membrane contactors and electrodialysis for ammonium recovery and concentration as a liquid fertilizer. *Chemosphere* **2020**, *245*, 125606.

(15) Li, Y.; Wang, R.; Shi, S.; Cao, H.; Yip, N. Y.; Lin, S. Bipolar Membrane Electrodialysis for Ammonia Recovery from Synthetic Urine: Experiments, Modeling, and Performance Analysis. *Environmental Science & Technology* **2021**, *55*, 14886–14896, Publisher: American Chemical Society.

(16) Ward, A. J.; Arola, K.; Thompson Brewster, E.; Mehta, C. M.; Batstone, D. J. Nutrient recovery from wastewater through pilot scale electrodialysis. *Water Research* **2018**, *135*, 57–65.

(17) van Linden, N.; Spanjers, H.; van Lier, J. B. Application of dynamic current density for increased concentration factors and reduced energy consumption for concentrating ammonium by electrodialysis. *Water Research* **2019**, *163*, 114856.
